# 1-{3-[(4-Oxopiperidin-1-yl)carbon­yl]benzoyl}piperidin-4-one

**DOI:** 10.1107/S1600536810026681

**Published:** 2010-07-10

**Authors:** K. Rajesh, V. Vijayakumar, S. Sarveswari, T. Narasimhamurthy, Edward R. T. Tiekink

**Affiliations:** a Organic Chemistry Division, School of Advanced Sciences, VIT University, Vellore 632 014, India; b Materials Research Centre, Indian Institute of Science, Bengaluru 560012, India; c Department of Chemistry, University of Malaya, 50603 Kuala Lumpur, Malaysia

## Abstract

Two independent mol­ecules comprise the asymmetric unit in the title compound, C_18_H_20_N_2_O_4_. One of the mol­ecules exhibits disorder in one of its 4-piperidone rings, which is disposed over two orientations [site occupancy of the major component = 0.651 (5)]. The first independent mol­ecule and the minor component of the second disordered mol­ecule are virtually superimposable. The central four C atoms in the major component of the disordered mol­ecule have an opposite orientation. All the 4-piperidone rings have a chair conformation. The carbonyl groups in each mol­ecule have approximate *anti* conformations [O=C⋯C=O = 146.2 (2) and −159.9 (2)°]. The 4-piperidone rings lie to opposite sides of the central benzene ring in both mol­ecules. In the crystal, mol­ecules are linked by C—H⋯O inter­actions. The crystal studied was found to be a non-merohedral twin (twin law −1 0 0, 0 1 0, 0 − 1/2 − 1), the fractional contribution of the minor component being approximately 11%.

## Related literature

For the background on the use of *N*-substituted-4-piperidones in organic synthesis, see: Dyakov *et al.* (1991 ▶); Scherer *et al.* (1993 ▶). For related structures, see: Vijayakumar *et al.* (2010 ▶); Rajesh *et al.* (2010 ▶).
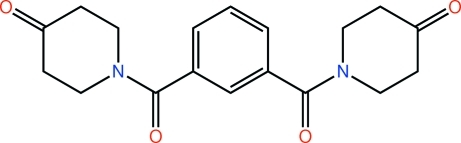


## Experimental

### 

#### Crystal data

C_18_H_20_N_2_O_4_
*M* *_r_* = 328.36
Triclinic, 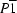

*a* = 10.777 (5) Å
*b* = 11.244 (5) Å
*c* = 13.665 (5) Å
α = 101.500 (5)°
β = 92.279 (5)°
γ = 90.009 (5)°
*V* = 1621.3 (12) Å^3^
*Z* = 4
Mo *K*α radiation
μ = 0.10 mm^−1^
*T* = 293 K
0.27 × 0.21 × 0.16 mm

#### Data collection

Bruker SMART APEX CCD diffractometer
33080 measured reflections
6729 independent reflections
4023 reflections with *I* > 2σ(*I*)
*R*_int_ = 0.060

#### Refinement

*R*[*F* ^2^ > 2σ(*F* ^2^)] = 0.054
*wR*(*F* ^2^) = 0.157
*S* = 1.05
6729 reflections
451 parameters
12 restraints
H-atom parameters constrained
Δρ_max_ = 0.32 e Å^−3^
Δρ_min_ = −0.32 e Å^−3^

### 

Data collection: *SMART* (Bruker, 2001 ▶); cell refinement: *SAINT* (Bruker, 2001 ▶); data reduction: *SAINT*; program(s) used to solve structure: *SHELXS97* (Sheldrick, 2008 ▶); program(s) used to refine structure: *SHELXL97* (Sheldrick, 2008 ▶) and *PLATON* (Spek, 2009 ▶); molecular graphics: *ORTEP-3* (Farrugia, 1997 ▶); software used to prepare material for publication: *publCIF* (Westrip, 2010 ▶).

## Supplementary Material

Crystal structure: contains datablocks global, I. DOI: 10.1107/S1600536810026681/hb5532sup1.cif

Structure factors: contains datablocks I. DOI: 10.1107/S1600536810026681/hb5532Isup2.hkl

Additional supplementary materials:  crystallographic information; 3D view; checkCIF report

## Figures and Tables

**Table 1:** Hydrogen-bond geometry (Å, °)

*D*—H⋯*A*	*D*—H	H⋯*A*	*D*⋯*A*	*D*—H⋯*A*
C4—H4b⋯O1^i^	0.97	2.58	3.383 (4)	141
C22—H22b⋯O5^ii^	0.97	2.44	3.295 (4)	146
C35—H35b⋯O8^iii^	0.97	2.55	3.297 (5)	134

Symmetry codes: (i) 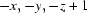; (ii) 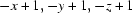; (iii) 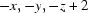.

**Fig. 1.**
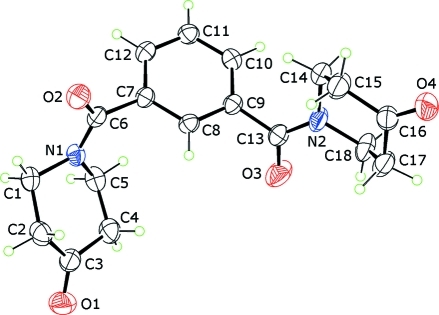
The molecular structure of first independent molecule, showing displacement ellipsoids at the 35% probability level.

**Fig. 2.**
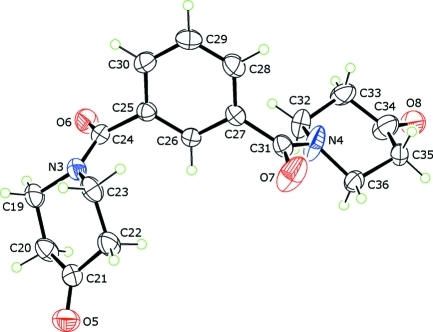
The molecular structure of second independent molecule, showing displacement ellipsoids at the 35% probability level. Only the major component of the disordered 4-piperidone residue is shown for reasons of clarity.

**Fig. 3.**
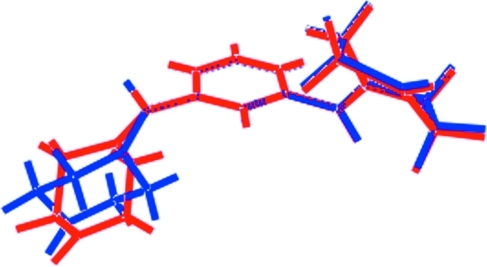
Overlay diagram of the first independent molecule with the inverted disordered molecule (minor component of the disordered molecule excluded).

**Fig. 4.**
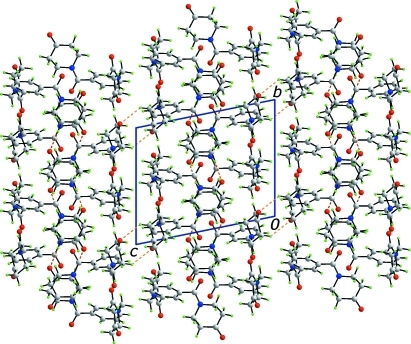
Stacking of supramolecular layers along the c axis in (I). The C–H···O contacts are shown as orange dashed lines.

## supplementary crystallographic information

### Comment

The synthesis of *N*-substituted-4-piperidones is subject of continuing interest owing to their importance as synthetic building blocks in medicinal chemistry, in particular for the synthesis of pharmacologically active agents (Dyakov *et al.*, 1991; Scherer *et al.*, 1993). In continuation of recent structural studies on *N*-substituted-4-piperidones (Vijayakumar *et al.*, 2010, Rajesh *et al.*, 2010), the title compound, (I), was investigated.

Two independent molecules comprise the asymmetric unit of (I). One molecule is ordered, Fig. 1, and the other is disordered, Fig. 2. In the disordered molecule, one 4-piperidone ring is disordered over two positions. The major component of the disorder has the central four carbon atoms of the N4-bound 4-piperidone ring in an opposite orientation to that found in the ordered molecule. This is emphasized in Fig. 3 which shows the superimposition of the ordered molecule upon the inverted disordered molecule. The minor component of the disordered ring has a conformation similar to that observed in the ordered molecule, Fig. 1. All 4-piperidone rings have a chair conformation. The carbonyl groups bound to the central benzene ring are almost *anti* as seen in the O═ C···C═O torsion angles of 146.2 (2) and -159.9 (2) ° for the two independent molecules, respectively. In each case, the 4-piperidone rings lie to opposite sides of the central benzene ring.

The most significant intermolecular contacts in the crystal structure are of the type C–H···O, Table 1, and these consolidate the crystal packing, Fig. 4.

### Experimental

To a suspension of 4-piperidone hydrochloride monohydrate (1 mmol) in benzene (20 ml) was added triethyl amine (3 mmol), followed by thorough stirring for 15 min. To that, isophthaloyl dichloride (0.5 mmol) dissolved in benzene (20 ml) was added drop wise with stirring, followed by refluxing for 7 h. The progress of the reaction was monitored by TLC. After the completion of the reaction, excess solvent was removed under reduced pressure. The crude products obtained were purified by column chromatography using an ethyl acetate/petroleum ether mixture (1:1). The sample (50 mg) was crystallized in 1:1 mixture of chloroform/methanol (5 + 5 ml) to yield colourless blocks of (I); m.pt. 415–417 K.

### Refinement

The C-bound H atoms were geometrically placed (C–H = 0.93–0.97 Å) and refined as riding with *U_iso_*(H) = 1.2*U_eq_*(C). Disorder in the N4-bound 4-piperidone ring was resolved over two positions. The major component (anisotropic refinement) had a site occupancy factor = 0.651 (5); the minor component was refined isotropically. The N–C and C–C distances in the disordered rings were refined with distance restraints 1.46±0.005 and 1.51±0.005 Å, respectively. For the treatment of twinned diffraction data, see: Spek (2009).

### Crystal data

**Table:**

C_18_H_20_N_2_O_4_	*Z* = 4
*M**_r_* = 328.36	*F*(000) = 696
Triclinic, *P*1	*D*_x_ = 1.345 Mg m^−^^3^
Hall symbol: -P 1	Mo *K*α radiation, λ = 0.71073 Å
*a* = 10.777 (5) Å	Cell parameters from 5023 reflections
*b* = 11.244 (5) Å	θ = 1.2–23.5°
*c* = 13.665 (5) Å	µ = 0.10 mm^−^^1^
α = 101.500 (5)°	*T* = 293 K
β = 92.279 (5)°	Block, colourless
γ = 90.009 (5)°	0.27 × 0.21 × 0.16 mm
*V* = 1621.3 (12) Å^3^	

### Data collection

**Table:**

Bruker SMART APEX CCD diffractometer	4023 reflections with *I* > 2σ(*I*)
Radiation source: fine-focus sealed tube	*R*_int_ = 0.060
graphite	θ_max_ = 26.5°, θ_min_ = 2.4°
ω scans	*h* = −13→13
33080 measured reflections	*k* = −14→14
6729 independent reflections	*l* = −17→17

### Refinement

**Table:**

Refinement on *F*^2^	Primary atom site location: structure-invariant direct methods
Least-squares matrix: full	Secondary atom site location: difference Fourier map
*R*[*F*^2^ > 2σ(*F*^2^)] = 0.054	Hydrogen site location: inferred from neighbouring sites
*wR*(*F*^2^) = 0.157	H-atom parameters constrained
*S* = 1.05	*w* = 1/[σ^2^(*F*_o_^2^) + (0.0747*P*)^2^ + 0.149*P*] where *P* = (*F*_o_^2^ + 2*F*_c_^2^)/3
6729 reflections	(Δ/σ)_max_ = 0.001
451 parameters	Δρ_max_ = 0.32 e Å^−^^3^
12 restraints	Δρ_min_ = −0.32 e Å^−^^3^

### Special details

**Table:**

Geometry. All s.u.'s (except the s.u. in the dihedral angle between two l.s. planes) are estimated using the full covariance matrix. The cell s.u.'s are taken into account individually in the estimation of s.u.'s in distances, angles and torsion angles; correlations between s.u.'s in cell parameters are only used when they are defined by crystal symmetry. An approximate (isotropic) treatment of cell s.u.'s is used for estimating s.u.'s involving l.s. planes.
Refinement. Refinement of *F*^2^ against ALL reflections. The weighted *R*-factor *wR* and goodness of fit *S* are based on *F*^2^, conventional *R*-factors *R* are based on *F*, with *F* set to zero for negative *F*^2^. The threshold expression of *F*^2^ > 2σ(*F*^2^) is used only for calculating *R*-factors(gt) *etc*. and is not relevant to the choice of reflections for refinement. *R*-factors based on *F*^2^ are statistically about twice as large as those based on *F*, and *R*- factors based on ALL data will be even larger.

### Fractional atomic coordinates and isotropic or equivalent isotropic displacement parameters (Å^2^)

**Table:**

	*x*	*y*	*z*	*U*_iso_*/*U*_eq_	Occ. (<1)
O1	0.0517 (2)	0.05185 (19)	0.37691 (15)	0.0823 (6)	
O2	0.15121 (18)	0.57298 (16)	0.53412 (13)	0.0638 (5)	
O3	−0.17857 (18)	0.34740 (19)	0.82228 (18)	0.0876 (7)	
O4	−0.55985 (18)	0.7338 (2)	0.87389 (15)	0.0817 (6)	
N1	0.17700 (18)	0.37391 (18)	0.53706 (13)	0.0467 (5)	
N2	−0.25096 (19)	0.53525 (19)	0.86990 (16)	0.0571 (6)	
C1	0.2050 (2)	0.3415 (2)	0.43153 (17)	0.0558 (7)	
H1A	0.2861	0.3040	0.4247	0.067*	
H1B	0.2069	0.4141	0.4034	0.067*	
C2	0.1071 (3)	0.2538 (2)	0.37500 (18)	0.0587 (7)	
H2A	0.0317	0.2985	0.3668	0.070*	
H2B	0.1360	0.2207	0.3089	0.070*	
C3	0.0769 (2)	0.1518 (2)	0.4244 (2)	0.0539 (6)	
C4	0.0729 (3)	0.1818 (2)	0.53700 (18)	0.0604 (7)	
H4A	−0.0073	0.2165	0.5557	0.072*	
H4B	0.0813	0.1076	0.5626	0.072*	
C5	0.1739 (2)	0.2699 (2)	0.58474 (18)	0.0524 (6)	
H5A	0.1592	0.2969	0.6552	0.063*	
H5B	0.2536	0.2295	0.5789	0.063*	
C6	0.1487 (2)	0.4878 (2)	0.57888 (17)	0.0441 (6)	
C7	0.1117 (2)	0.5125 (2)	0.68628 (16)	0.0403 (5)	
C8	−0.0031 (2)	0.4751 (2)	0.71243 (16)	0.0421 (5)	
H8	−0.0558	0.4292	0.6637	0.050*	
C9	−0.0401 (2)	0.5051 (2)	0.80980 (16)	0.0408 (5)	
C10	0.0404 (2)	0.5695 (2)	0.88251 (17)	0.0465 (6)	
H10	0.0166	0.5893	0.9484	0.056*	
C11	0.1561 (2)	0.6045 (2)	0.85785 (18)	0.0485 (6)	
H11	0.2106	0.6462	0.9074	0.058*	
C12	0.1907 (2)	0.5776 (2)	0.75993 (17)	0.0452 (6)	
H12	0.2677	0.6035	0.7433	0.054*	
C13	−0.1622 (2)	0.4571 (2)	0.83514 (17)	0.0469 (6)	
C14	−0.2436 (2)	0.6670 (2)	0.8809 (2)	0.0612 (7)	
H14A	−0.1656	0.6890	0.8559	0.073*	
H14B	−0.2453	0.7046	0.9512	0.073*	
C15	−0.3504 (2)	0.7144 (3)	0.8245 (2)	0.0665 (8)	
H15A	−0.3500	0.8024	0.8411	0.080*	
H15B	−0.3393	0.6898	0.7533	0.080*	
C16	−0.4735 (2)	0.6676 (3)	0.84948 (19)	0.0586 (7)	
C17	−0.4781 (2)	0.5332 (3)	0.8422 (2)	0.0632 (7)	
H17A	−0.4755	0.4926	0.7726	0.076*	
H17B	−0.5554	0.5108	0.8681	0.076*	
C18	−0.3685 (2)	0.4922 (3)	0.9015 (2)	0.0671 (8)	
H18A	−0.3772	0.5240	0.9722	0.081*	
H18B	−0.3681	0.4043	0.8910	0.081*	
O5	0.5437 (2)	0.38661 (19)	0.38183 (15)	0.0794 (6)	
O6	0.64560 (18)	−0.05160 (16)	0.54295 (13)	0.0646 (5)	
O7	0.2983 (2)	0.31306 (19)	0.8416 (2)	0.1035 (8)	
O8	−0.08234 (17)	−0.06160 (18)	0.88350 (15)	0.0737 (6)	
N3	0.67154 (18)	0.14907 (18)	0.54821 (13)	0.0481 (5)	
C19	0.7071 (3)	0.1321 (3)	0.44466 (18)	0.0589 (7)	
H19A	0.7131	0.0461	0.4167	0.071*	
H19B	0.7880	0.1689	0.4420	0.071*	
C20	0.6126 (3)	0.1891 (2)	0.38345 (18)	0.0578 (7)	
H20A	0.6475	0.1930	0.3198	0.069*	
H20B	0.5399	0.1367	0.3699	0.069*	
C21	0.5730 (2)	0.3124 (2)	0.43124 (18)	0.0510 (6)	
C22	0.5636 (3)	0.3373 (2)	0.54298 (18)	0.0599 (7)	
H22A	0.4829	0.3101	0.5591	0.072*	
H22B	0.5693	0.4242	0.5681	0.072*	
C23	0.6631 (2)	0.2758 (2)	0.59541 (18)	0.0539 (6)	
H23A	0.7424	0.3157	0.5930	0.065*	
H23B	0.6438	0.2826	0.6650	0.065*	
C24	0.6393 (2)	0.0544 (2)	0.58790 (17)	0.0446 (6)	
C25	0.5937 (2)	0.0812 (2)	0.69292 (16)	0.0402 (5)	
C26	0.4768 (2)	0.1274 (2)	0.71318 (16)	0.0413 (5)	
H26	0.4265	0.1460	0.6615	0.050*	
C27	0.4329 (2)	0.1466 (2)	0.80919 (16)	0.0419 (5)	
C28	0.5098 (3)	0.1197 (3)	0.88504 (18)	0.0594 (7)	
H28	0.4825	0.1326	0.9500	0.071*	
C29	0.6268 (3)	0.0741 (3)	0.86509 (19)	0.0660 (8)	
H29	0.6780	0.0568	0.9167	0.079*	
C30	0.6685 (2)	0.0539 (2)	0.76915 (19)	0.0545 (6)	
H30	0.7470	0.0219	0.7560	0.065*	
C31	0.3084 (2)	0.2047 (2)	0.82956 (16)	0.0462 (6)	
N4	0.2093 (2)	0.13604 (18)	0.8326 (2)	0.0849 (9)	0.651 (5)
C32	0.2036 (4)	0.0042 (3)	0.7921 (3)	0.0601 (13)	0.651 (5)
H32A	0.2866	−0.0286	0.7838	0.072*	0.651 (5)
H32B	0.1590	−0.0125	0.7278	0.072*	0.651 (5)
C33	0.1364 (4)	−0.0516 (4)	0.8672 (5)	0.0654 (15)	0.651 (5)
H33A	0.1330	−0.1392	0.8461	0.078*	0.651 (5)
H33B	0.1795	−0.0318	0.9322	0.078*	0.651 (5)
C34	0.0071 (2)	−0.0008 (2)	0.8733 (3)	0.0783 (9)	0.651 (5)
C35	0.0043 (3)	0.1382 (3)	0.9018 (3)	0.0468 (10)	0.651 (5)
H35A	−0.0805	0.1664	0.8990	0.056*	0.651 (5)
H35B	0.0385	0.1654	0.9693	0.056*	0.651 (5)
C36	0.0810 (3)	0.1890 (3)	0.8286 (4)	0.0525 (11)	0.651 (5)
H36A	0.0439	0.1667	0.7616	0.063*	0.651 (5)
H36B	0.0852	0.2769	0.8471	0.063*	0.651 (5)
N4'	0.2093 (2)	0.13604 (18)	0.8326 (2)	0.0849 (9)	0.349 (5)
C32'	0.2315 (6)	0.0198 (5)	0.8719 (6)	0.051 (2)*	0.349 (5)
H32C	0.2297	0.0347	0.9442	0.061*	0.349 (5)
H32D	0.3102	−0.0165	0.8511	0.061*	0.349 (5)
C33'	0.1227 (5)	−0.0600 (9)	0.8235 (7)	0.059 (3)*	0.349 (5)
H33C	0.1322	−0.1420	0.8350	0.071*	0.349 (5)
H33D	0.1171	−0.0629	0.7520	0.071*	0.349 (5)
C34'	0.0071 (2)	−0.0008 (2)	0.8733 (3)	0.0783 (9)	0.349 (5)
C35'	−0.0040 (7)	0.1174 (5)	0.8348 (6)	0.061 (2)*	0.349 (5)
H35C	0.0037	0.1031	0.7630	0.073*	0.349 (5)
H35D	−0.0827	0.1566	0.8517	0.073*	0.349 (5)
C36'	0.1034 (5)	0.1925 (7)	0.8885 (6)	0.059 (3)*	0.349 (5)
H36C	0.0953	0.2772	0.8841	0.071*	0.349 (5)
H36D	0.1109	0.1861	0.9582	0.071*	0.349 (5)

### Atomic displacement parameters (Å^2^)

**Table:**

	*U*^11^	*U*^22^	*U*^33^	*U*^12^	*U*^13^	*U*^23^
O1	0.1075 (17)	0.0681 (14)	0.0680 (13)	−0.0254 (12)	0.0079 (12)	0.0048 (11)
O2	0.0859 (14)	0.0568 (11)	0.0543 (11)	−0.0098 (10)	0.0125 (10)	0.0228 (9)
O3	0.0683 (13)	0.0556 (13)	0.144 (2)	−0.0076 (10)	0.0246 (13)	0.0285 (12)
O4	0.0487 (11)	0.0999 (16)	0.0840 (15)	0.0016 (11)	0.0113 (10)	−0.0130 (12)
N1	0.0523 (12)	0.0520 (13)	0.0373 (11)	0.0008 (9)	0.0084 (9)	0.0115 (9)
N2	0.0468 (12)	0.0563 (14)	0.0728 (15)	−0.0048 (10)	0.0209 (11)	0.0207 (11)
C1	0.0608 (16)	0.0644 (17)	0.0439 (14)	−0.0018 (13)	0.0158 (12)	0.0121 (12)
C2	0.0723 (18)	0.0638 (17)	0.0410 (14)	0.0021 (14)	0.0075 (13)	0.0116 (12)
C3	0.0506 (15)	0.0531 (16)	0.0587 (16)	0.0003 (12)	0.0070 (12)	0.0119 (13)
C4	0.0790 (19)	0.0534 (16)	0.0534 (16)	0.0028 (14)	0.0176 (14)	0.0190 (12)
C5	0.0588 (15)	0.0574 (16)	0.0448 (14)	0.0175 (13)	0.0092 (12)	0.0181 (12)
C6	0.0375 (12)	0.0536 (15)	0.0437 (13)	−0.0081 (11)	0.0028 (10)	0.0157 (12)
C7	0.0413 (12)	0.0415 (13)	0.0405 (13)	−0.0006 (10)	0.0021 (10)	0.0135 (10)
C8	0.0400 (12)	0.0466 (13)	0.0400 (13)	−0.0044 (10)	−0.0014 (10)	0.0101 (10)
C9	0.0385 (12)	0.0412 (13)	0.0444 (13)	0.0025 (10)	0.0023 (10)	0.0128 (10)
C10	0.0505 (14)	0.0484 (14)	0.0411 (13)	0.0033 (11)	0.0055 (11)	0.0090 (11)
C11	0.0443 (14)	0.0492 (14)	0.0492 (15)	−0.0035 (11)	−0.0048 (11)	0.0044 (11)
C12	0.0364 (12)	0.0477 (14)	0.0530 (15)	−0.0025 (10)	0.0016 (11)	0.0136 (11)
C13	0.0472 (14)	0.0481 (15)	0.0490 (14)	−0.0042 (12)	0.0058 (11)	0.0173 (11)
C14	0.0492 (15)	0.0595 (17)	0.0718 (18)	−0.0083 (13)	0.0164 (13)	0.0027 (13)
C15	0.0512 (16)	0.0593 (17)	0.091 (2)	−0.0015 (13)	0.0156 (15)	0.0179 (15)
C16	0.0463 (15)	0.076 (2)	0.0489 (15)	−0.0043 (14)	0.0039 (12)	0.0019 (13)
C17	0.0489 (15)	0.081 (2)	0.0613 (17)	−0.0182 (14)	0.0103 (13)	0.0177 (14)
C18	0.0530 (16)	0.082 (2)	0.0747 (19)	−0.0085 (14)	0.0232 (14)	0.0314 (16)
O5	0.1040 (16)	0.0772 (14)	0.0626 (12)	0.0314 (12)	0.0150 (11)	0.0252 (11)
O6	0.0862 (14)	0.0492 (11)	0.0583 (11)	0.0143 (9)	0.0210 (10)	0.0069 (9)
O7	0.0663 (14)	0.0498 (13)	0.199 (3)	0.0023 (10)	0.0305 (15)	0.0313 (14)
O8	0.0566 (12)	0.0771 (14)	0.0938 (15)	−0.0121 (10)	0.0025 (10)	0.0324 (11)
N3	0.0531 (12)	0.0516 (12)	0.0400 (11)	0.0026 (9)	0.0106 (9)	0.0086 (9)
C19	0.0681 (17)	0.0643 (17)	0.0466 (15)	0.0132 (14)	0.0225 (13)	0.0127 (12)
C20	0.0815 (19)	0.0520 (16)	0.0411 (14)	0.0001 (14)	0.0112 (13)	0.0102 (11)
C21	0.0489 (14)	0.0557 (16)	0.0509 (15)	0.0004 (12)	0.0082 (12)	0.0155 (12)
C22	0.0818 (19)	0.0497 (15)	0.0492 (15)	0.0076 (14)	0.0195 (14)	0.0089 (12)
C23	0.0640 (16)	0.0520 (15)	0.0448 (14)	−0.0178 (13)	0.0076 (12)	0.0062 (11)
C24	0.0390 (12)	0.0508 (15)	0.0448 (14)	0.0072 (11)	0.0037 (10)	0.0106 (12)
C25	0.0404 (12)	0.0403 (13)	0.0404 (13)	−0.0025 (10)	0.0002 (10)	0.0096 (10)
C26	0.0426 (13)	0.0470 (13)	0.0351 (12)	0.0016 (10)	−0.0016 (10)	0.0106 (10)
C27	0.0459 (13)	0.0430 (13)	0.0365 (13)	−0.0039 (10)	0.0032 (10)	0.0070 (10)
C28	0.0635 (17)	0.0771 (19)	0.0380 (14)	0.0008 (14)	0.0033 (12)	0.0123 (12)
C29	0.0628 (18)	0.092 (2)	0.0453 (16)	0.0058 (16)	−0.0132 (13)	0.0218 (14)
C30	0.0454 (14)	0.0657 (17)	0.0528 (16)	0.0063 (12)	−0.0035 (12)	0.0141 (12)
C31	0.0534 (15)	0.0453 (15)	0.0393 (13)	−0.0015 (12)	0.0091 (11)	0.0056 (10)
N4	0.0552 (14)	0.0385 (13)	0.161 (3)	0.0060 (10)	0.0509 (16)	0.0108 (14)
C32	0.047 (2)	0.053 (3)	0.072 (3)	−0.0023 (18)	0.016 (2)	−0.010 (2)
C33	0.063 (3)	0.047 (3)	0.092 (4)	0.005 (2)	0.003 (3)	0.027 (3)
C34	0.0522 (17)	0.067 (2)	0.126 (3)	0.0006 (15)	0.0085 (17)	0.0418 (19)
C35	0.042 (2)	0.054 (2)	0.045 (2)	0.0069 (17)	0.0108 (17)	0.0095 (17)
C36	0.051 (2)	0.048 (2)	0.064 (3)	0.0118 (18)	0.016 (2)	0.022 (2)
N4'	0.0552 (14)	0.0385 (13)	0.161 (3)	0.0060 (10)	0.0509 (16)	0.0108 (14)
C34'	0.0522 (17)	0.067 (2)	0.126 (3)	0.0006 (15)	0.0085 (17)	0.0418 (19)

### Geometric parameters (Å, °)

**Table:**

O1—C3	1.205 (3)	C19—C20	1.514 (4)
O2—C6	1.236 (3)	C19—H19A	0.9700
O3—C13	1.222 (3)	C19—H19B	0.9700
O4—C16	1.207 (3)	C20—C21	1.481 (3)
N1—C6	1.335 (3)	C20—H20A	0.9700
N1—C5	1.449 (3)	C20—H20B	0.9700
N1—C1	1.459 (3)	C21—C22	1.504 (3)
N2—C13	1.335 (3)	C22—C23	1.510 (4)
N2—C14	1.461 (3)	C22—H22A	0.9700
N2—C18	1.467 (3)	C22—H22B	0.9700
C1—C2	1.521 (4)	C23—H23A	0.9700
C1—H1A	0.9700	C23—H23B	0.9700
C1—H1B	0.9700	C24—C25	1.509 (3)
C2—C3	1.484 (4)	C25—C30	1.375 (3)
C2—H2A	0.9700	C25—C26	1.379 (3)
C2—H2B	0.9700	C26—C27	1.389 (3)
C3—C4	1.510 (4)	C26—H26	0.9300
C4—C5	1.509 (4)	C27—C28	1.382 (3)
C4—H4A	0.9700	C27—C31	1.504 (3)
C4—H4B	0.9700	C28—C29	1.377 (4)
C5—H5A	0.9700	C28—H28	0.9300
C5—H5B	0.9700	C29—C30	1.379 (3)
C6—C7	1.508 (3)	C29—H29	0.9300
C7—C12	1.382 (3)	C30—H30	0.9300
C7—C8	1.390 (3)	C31—N4'	1.324 (3)
C8—C9	1.381 (3)	C31—N4	1.324 (3)
C8—H8	0.9300	N4—C32	1.476 (3)
C9—C10	1.381 (3)	N4—C36	1.509 (3)
C9—C13	1.501 (3)	C32—C33	1.513 (4)
C10—C11	1.381 (3)	C32—H32A	0.9700
C10—H10	0.9300	C32—H32B	0.9700
C11—C12	1.378 (3)	C33—C34	1.505 (4)
C11—H11	0.9300	C33—H33A	0.9700
C12—H12	0.9300	C33—H33B	0.9700
C14—C15	1.516 (4)	C34—C35	1.534 (4)
C14—H14A	0.9700	C35—C36	1.517 (4)
C14—H14B	0.9700	C35—H35A	0.9700
C15—C16	1.504 (4)	C35—H35B	0.9700
C15—H15A	0.9700	C36—H36A	0.9700
C15—H15B	0.9700	C36—H36B	0.9700
C16—C17	1.495 (4)	N4'—C36'	1.471 (5)
C17—C18	1.530 (4)	N4'—C32'	1.524 (4)
C17—H17A	0.9700	C32'—C33'	1.522 (5)
C17—H17B	0.9700	C32'—H32C	0.9700
C18—H18A	0.9700	C32'—H32D	0.9700
C18—H18B	0.9700	C33'—C34'	1.532 (5)
O5—C21	1.208 (3)	C33'—H33C	0.9700
O6—C24	1.230 (3)	C33'—H33D	0.9700
O7—C31	1.202 (3)	C34'—C35'	1.526 (5)
O8—C34'	1.210 (3)	C35'—C36'	1.509 (5)
O8—C34	1.210 (3)	C35'—H35C	0.9700
N3—C24	1.339 (3)	C35'—H35D	0.9700
N3—C23	1.446 (3)	C36'—H36C	0.9700
N3—C19	1.456 (3)	C36'—H36D	0.9700
			
C6—N1—C5	126.11 (19)	O5—C21—C22	122.1 (2)
C6—N1—C1	120.8 (2)	C20—C21—C22	116.6 (2)
C5—N1—C1	112.87 (19)	C21—C22—C23	113.4 (2)
C13—N2—C14	125.6 (2)	C21—C22—H22A	108.9
C13—N2—C18	120.7 (2)	C23—C22—H22A	108.9
C14—N2—C18	113.6 (2)	C21—C22—H22B	108.9
N1—C1—C2	110.16 (19)	C23—C22—H22B	108.9
N1—C1—H1A	109.6	H22A—C22—H22B	107.7
C2—C1—H1A	109.6	N3—C23—C22	110.4 (2)
N1—C1—H1B	109.6	N3—C23—H23A	109.6
C2—C1—H1B	109.6	C22—C23—H23A	109.6
H1A—C1—H1B	108.1	N3—C23—H23B	109.6
C3—C2—C1	114.2 (2)	C22—C23—H23B	109.6
C3—C2—H2A	108.7	H23A—C23—H23B	108.1
C1—C2—H2A	108.7	O6—C24—N3	123.1 (2)
C3—C2—H2B	108.7	O6—C24—C25	119.5 (2)
C1—C2—H2B	108.7	N3—C24—C25	117.4 (2)
H2A—C2—H2B	107.6	C30—C25—C26	119.5 (2)
O1—C3—C2	121.7 (2)	C30—C25—C24	119.0 (2)
O1—C3—C4	122.0 (2)	C26—C25—C24	121.44 (19)
C2—C3—C4	116.3 (2)	C25—C26—C27	121.3 (2)
C5—C4—C3	112.7 (2)	C25—C26—H26	119.4
C5—C4—H4A	109.1	C27—C26—H26	119.4
C3—C4—H4A	109.1	C28—C27—C26	118.4 (2)
C5—C4—H4B	109.1	C28—C27—C31	121.8 (2)
C3—C4—H4B	109.1	C26—C27—C31	119.7 (2)
H4A—C4—H4B	107.8	C29—C28—C27	120.4 (2)
N1—C5—C4	110.7 (2)	C29—C28—H28	119.8
N1—C5—H5A	109.5	C27—C28—H28	119.8
C4—C5—H5A	109.5	C28—C29—C30	120.5 (2)
N1—C5—H5B	109.5	C28—C29—H29	119.7
C4—C5—H5B	109.5	C30—C29—H29	119.7
H5A—C5—H5B	108.1	C25—C30—C29	119.9 (2)
O2—C6—N1	123.4 (2)	C25—C30—H30	120.1
O2—C6—C7	118.9 (2)	C29—C30—H30	120.1
N1—C6—C7	117.74 (19)	O7—C31—N4'	119.9 (2)
C12—C7—C8	119.0 (2)	O7—C31—N4	119.9 (2)
C12—C7—C6	119.9 (2)	O7—C31—C27	120.2 (2)
C8—C7—C6	121.05 (19)	N4'—C31—C27	119.9 (2)
C9—C8—C7	120.9 (2)	N4—C31—C27	119.9 (2)
C9—C8—H8	119.5	C31—N4—C32	123.9 (2)
C7—C8—H8	119.5	C31—N4—C36	120.1 (2)
C8—C9—C10	119.2 (2)	C32—N4—C36	109.7 (3)
C8—C9—C13	118.8 (2)	N4—C32—C33	106.3 (3)
C10—C9—C13	121.8 (2)	N4—C32—H32A	110.5
C9—C10—C11	120.4 (2)	C33—C32—H32A	110.5
C9—C10—H10	119.8	N4—C32—H32B	110.5
C11—C10—H10	119.8	C33—C32—H32B	110.5
C12—C11—C10	120.1 (2)	H32A—C32—H32B	108.7
C12—C11—H11	120.0	C34—C33—C32	107.6 (3)
C10—C11—H11	120.0	C34—C33—H33A	110.2
C11—C12—C7	120.4 (2)	C32—C33—H33A	110.2
C11—C12—H12	119.8	C34—C33—H33B	110.2
C7—C12—H12	119.8	C32—C33—H33B	110.2
O3—C13—N2	121.6 (2)	H33A—C33—H33B	108.5
O3—C13—C9	119.2 (2)	O8—C34—C33	122.2 (3)
N2—C13—C9	119.2 (2)	O8—C34—C35	121.2 (3)
N2—C14—C15	111.3 (2)	C33—C34—C35	113.4 (3)
N2—C14—H14A	109.4	C36—C35—C34	108.4 (3)
C15—C14—H14A	109.4	C36—C35—H35A	110.0
N2—C14—H14B	109.4	C34—C35—H35A	110.0
C15—C14—H14B	109.4	C36—C35—H35B	110.0
H14A—C14—H14B	108.0	C34—C35—H35B	110.0
C16—C15—C14	111.7 (2)	H35A—C35—H35B	108.4
C16—C15—H15A	109.3	N4—C36—C35	107.2 (3)
C14—C15—H15A	109.3	N4—C36—H36A	110.3
C16—C15—H15B	109.3	C35—C36—H36A	110.3
C14—C15—H15B	109.3	N4—C36—H36B	110.3
H15A—C15—H15B	107.9	C35—C36—H36B	110.3
O4—C16—C17	123.7 (3)	H36A—C36—H36B	108.5
O4—C16—C15	122.3 (3)	C31—N4'—C36'	117.8 (3)
C17—C16—C15	114.0 (2)	C31—N4'—C32'	116.2 (3)
C16—C17—C18	110.4 (2)	C36'—N4'—C32'	104.0 (5)
C16—C17—H17A	109.6	N4'—C32'—C33'	102.3 (6)
C18—C17—H17A	109.6	N4'—C32'—H32C	111.3
C16—C17—H17B	109.6	C33'—C32'—H32C	111.3
C18—C17—H17B	109.6	N4'—C32'—H32D	111.3
H17A—C17—H17B	108.1	C33'—C32'—H32D	111.3
N2—C18—C17	110.5 (2)	H32C—C32'—H32D	109.2
N2—C18—H18A	109.6	C32'—C33'—C34'	105.5 (5)
C17—C18—H18A	109.6	C32'—C33'—H33C	110.6
N2—C18—H18B	109.6	C34'—C33'—H33C	110.6
C17—C18—H18B	109.6	C32'—C33'—H33D	110.6
H18A—C18—H18B	108.1	C34'—C33'—H33D	110.6
C24—N3—C23	126.11 (19)	H33C—C33'—H33D	108.8
C24—N3—C19	121.0 (2)	O8—C34'—C35'	122.6 (4)
C23—N3—C19	112.53 (19)	O8—C34'—C33'	120.7 (4)
N3—C19—C20	110.6 (2)	C35'—C34'—C33'	103.4 (6)
N3—C19—H19A	109.5	C36'—C35'—C34'	103.0 (5)
C20—C19—H19A	109.5	C36'—C35'—H35C	111.2
N3—C19—H19B	109.5	C34'—C35'—H35C	111.2
C20—C19—H19B	109.5	C36'—C35'—H35D	111.2
H19A—C19—H19B	108.1	C34'—C35'—H35D	111.2
C21—C20—C19	114.7 (2)	H35C—C35'—H35D	109.1
C21—C20—H20A	108.6	N4'—C36'—C35'	101.6 (5)
C19—C20—H20A	108.6	N4'—C36'—H36C	111.4
C21—C20—H20B	108.6	C35'—C36'—H36C	111.4
C19—C20—H20B	108.6	N4'—C36'—H36D	111.4
H20A—C20—H20B	107.6	C35'—C36'—H36D	111.4
O5—C21—C20	121.2 (2)	H36C—C36'—H36D	109.3
			
C6—N1—C1—C2	−115.3 (2)	C19—N3—C23—C22	62.4 (3)
C5—N1—C1—C2	60.0 (3)	C21—C22—C23—N3	−48.9 (3)
N1—C1—C2—C3	−46.9 (3)	C23—N3—C24—O6	178.1 (2)
C1—C2—C3—O1	−145.0 (3)	C19—N3—C24—O6	5.1 (4)
C1—C2—C3—C4	38.2 (3)	C23—N3—C24—C25	−2.1 (3)
O1—C3—C4—C5	144.0 (3)	C19—N3—C24—C25	−175.1 (2)
C2—C3—C4—C5	−39.2 (3)	O6—C24—C25—C30	71.4 (3)
C6—N1—C5—C4	112.9 (3)	N3—C24—C25—C30	−108.4 (3)
C1—N1—C5—C4	−62.0 (3)	O6—C24—C25—C26	−105.7 (3)
C3—C4—C5—N1	49.6 (3)	N3—C24—C25—C26	74.5 (3)
C5—N1—C6—O2	−178.9 (2)	C30—C25—C26—C27	−0.2 (3)
C1—N1—C6—O2	−4.4 (3)	C24—C25—C26—C27	176.9 (2)
C5—N1—C6—C7	1.0 (3)	C25—C26—C27—C28	0.8 (3)
C1—N1—C6—C7	175.6 (2)	C25—C26—C27—C31	176.0 (2)
O2—C6—C7—C12	−70.4 (3)	C26—C27—C28—C29	−0.6 (4)
N1—C6—C7—C12	109.7 (2)	C31—C27—C28—C29	−175.6 (2)
O2—C6—C7—C8	107.0 (3)	C27—C28—C29—C30	−0.3 (4)
N1—C6—C7—C8	−72.9 (3)	C26—C25—C30—C29	−0.7 (4)
C12—C7—C8—C9	1.9 (3)	C24—C25—C30—C29	−177.9 (2)
C6—C7—C8—C9	−175.5 (2)	C28—C29—C30—C25	1.0 (4)
C7—C8—C9—C10	−2.4 (3)	C28—C27—C31—O7	94.2 (3)
C7—C8—C9—C13	−176.9 (2)	C26—C27—C31—O7	−80.7 (3)
C8—C9—C10—C11	0.7 (3)	C28—C27—C31—N4'	−86.9 (3)
C13—C9—C10—C11	175.0 (2)	C26—C27—C31—N4'	98.2 (3)
C9—C10—C11—C12	1.5 (4)	C28—C27—C31—N4	−86.9 (3)
C10—C11—C12—C7	−1.9 (4)	C26—C27—C31—N4	98.2 (3)
C8—C7—C12—C11	0.3 (3)	O7—C31—N4—C32	162.4 (3)
C6—C7—C12—C11	177.7 (2)	C27—C31—N4—C32	−16.6 (5)
C14—N2—C13—O3	−175.3 (3)	O7—C31—N4—C36	13.3 (5)
C18—N2—C13—O3	4.9 (4)	C27—C31—N4—C36	−165.6 (3)
C14—N2—C13—C9	3.8 (4)	C31—N4—C32—C33	138.5 (4)
C18—N2—C13—C9	−176.1 (2)	C36—N4—C32—C33	−69.7 (4)
C8—C9—C13—O3	60.7 (3)	N4—C32—C33—C34	62.4 (5)
C10—C9—C13—O3	−113.7 (3)	C32—C33—C34—O8	142.8 (4)
C8—C9—C13—N2	−118.4 (3)	C32—C33—C34—C35	−57.2 (5)
C10—C9—C13—N2	67.2 (3)	O8—C34—C35—C36	−145.0 (4)
C13—N2—C14—C15	123.8 (3)	C33—C34—C35—C36	54.7 (5)
C18—N2—C14—C15	−56.4 (3)	C31—N4—C36—C35	−139.9 (3)
N2—C14—C15—C16	50.7 (3)	C32—N4—C36—C35	67.1 (4)
C14—C15—C16—O4	129.6 (3)	C34—C35—C36—N4	−56.5 (4)
C14—C15—C16—C17	−49.9 (3)	O7—C31—N4'—C36'	−24.0 (6)
O4—C16—C17—C18	−128.2 (3)	C27—C31—N4'—C36'	157.1 (5)
C15—C16—C17—C18	51.3 (3)	O7—C31—N4'—C32'	−148.4 (4)
C13—N2—C18—C17	−122.2 (3)	C27—C31—N4'—C32'	32.7 (5)
C14—N2—C18—C17	58.0 (3)	C31—N4'—C32'—C33'	−155.4 (4)
C16—C17—C18—N2	−53.9 (3)	C36'—N4'—C32'—C33'	73.5 (6)
C24—N3—C19—C20	113.6 (2)	N4'—C32'—C33'—C34'	−68.4 (8)
C23—N3—C19—C20	−60.3 (3)	C32'—C33'—C34'—O8	−149.6 (5)
N3—C19—C20—C21	45.5 (3)	C32'—C33'—C34'—C35'	68.6 (8)
C19—C20—C21—O5	148.6 (3)	O8—C34'—C35'—C36'	148.0 (5)
C19—C20—C21—C22	−35.2 (3)	C33'—C34'—C35'—C36'	−71.1 (7)
O5—C21—C22—C23	−147.1 (3)	C31—N4'—C36'—C35'	152.4 (5)
C20—C21—C22—C23	36.7 (3)	C32'—N4'—C36'—C35'	−77.4 (7)
C24—N3—C23—C22	−111.1 (3)	C34'—C35'—C36'—N4'	75.8 (8)

### Hydrogen-bond geometry (Å, °)

**Table:**

*D*—H···*A*	*D*—H	H···*A*	*D*···*A*	*D*—H···*A*
C4—H4b···O1^i^	0.97	2.58	3.383 (4)	141
C22—H22b···O5^ii^	0.97	2.44	3.295 (4)	146
C35—H35b···O8^iii^	0.97	2.55	3.297 (5)	134

Symmetry codes: (i) −*x*, −*y*, −*z*+1; (ii) −*x*+1, −*y*+1, −*z*+1; (iii) −*x*, −*y*, −*z*+2.
